# Developing a computerised search to help UK General Practices identify more patients for palliative care planning: a feasibility study

**DOI:** 10.1186/s12875-015-0312-z

**Published:** 2015-08-08

**Authors:** Bruce Mason, Kirsty Boyd, Scott A Murray, John Steyn, Paul Cormie, Marilyn Kendall, Dan Munday, David Weller, Shirley Fife, Peter Murchie, Christine Campbell

**Affiliations:** Centre for Population Health Sciences, University of Edinburgh, Medical School, Teviot Place, Edinburgh, EH8 9AG UK; NHS Lothian, Edinburgh, Midlothian UK; NHS Borders, Melrose, Roxburghshire UK; Health Sciences, Medical School Building, Gibbet Hill Campus, University of Warwick, Coventry, CV4 UK; Centre of Academic Primary Care, Division of Applied Health Sciences, University of Aberdeen, Polwarth Building, Foresterhill, Aberdeen, AB25 2ZD UK

**Keywords:** Primary healthcare, General practice, Palliative care, Qualitative research

## Abstract

**Background:**

Approximately 600,000 people die in the UK annually, usually after months or years of increasing debility. Many patients with advanced conditions are not identified for appropriate support before they die because they are not seen as having “palliative” care needs. General practice information technology systems can improve care by identifying patients with deteriorating health so that their healthcare needs can be reviewed more systematically and effectively. The aim was to develop and test a computerised search of primary care records in routine clinical practice as a tool to improve patient identification for a palliative care approach.

**Methods:**

An iterative process of search design and testing followed by implementation and extended testing of the search output in clinical practice. A three-phase feasibility study: developing a computerised search, determining its ability to identify patients with deteriorating health from any advanced condition, and assessing how primary care clinicians use the results to improve patient care. The setting was twelve primary care teams in two Health Boards in Scotland.

**Results:**

The search identified 0.6–1.7 % of patients in each practice who were not already on the palliative care register. Primary care clinicians judged that 30–60 % of these patients were at risk of dying or deterioration over the next 6–12 months. The most common action taken by GPs was to start an electronic anticipatory care plan.

**Conclusions:**

It is possible to significantly improve the identification of patients for palliative care needs assessment using a computerised search however barriers remain to GPs’ finding it acceptable. Time-efficient systems were important as was a generic tool for anticipatory care planning not linked to ‘palliative’ care.

## Background

Approximately 600,000 people die in the UK each year, usually after months or years of increasing debility www.ons.gov.uk/ons/publications/index.html [1]. UK health policy emphasises early identification, assessment and care planning for these patients [2, 3], and the need for a greater evidence base to support the provision of palliative care in the community [4]. Although most patients spend the majority of their last year of life at home, around 50 % eventually die in hospital [5].

Most patients are not identified for a palliative care approach before they die [6] because they are not identified as having ‘palliative’ care needs [7, 8]. A study of six Scottish general practices in 2012, found that only 29 % of deceased patients were on the palliative care register [9]. Only 30 % of deaths overall were from cancer, but 68 % of patients on the palliative care register had cancer [9]. A review of deaths in high income countries indicated that between 69–82 % of patients who die need palliative care [10]. Unless practical guidance and tools are readily available to practices to support systematic patient identification, this inequality will continue.

Searching patient data held in general practice information technology (IT) systems offers a means to improve palliative care for patients and their families by helping primary care identify them and manage their healthcare needs more effectively [11, 12]. IT systems that fit with the clinical management systems in practices, and the values and priorities of GPs are the most successful [11, 13]. There has been interest in developing such tools; a recent systematic literature review across Europe to identify tools that support identification of patients with palliative care needs found seven but none was in widespread use [14].

Our central question, therefore, was to see whether it was possible to develop a simple electronic record search that required no additional software to implement and no specialist skills to use that could increase the number of patients with palliative care needs being identified in primary care. In addition, we explored whether such a search was acceptable to professionals involved. If the search was feasible and acceptable we believed it would facilitate the identification of patients for palliative care earlier in the illness and according to need, not diagnosis, so that more people with non-cancer illnesses could benefit from proactive care [15].

## Method

The participating primary care teams were in a mixed urban—rural and a largely urban Health Board in Scotland. Ethical review was conducted by the South East Scotland Research Ethics Committee (12/SS/0040) which gave a favourable opinion to proceed with this study as a service evaluation. The study had three phases over 18 months, starting in 2013.

### Phase 1): development of the search algorithm

We developed a computerised search based on Read codes [16] that could be run in “Vision”: the most common software used in general practices in Scotland (http://www.inps4.co.uk/vision/vision-your-region). The search did not require any additional software programming, coding of new information or additional user-expertise beyond the ability to run patient record searches. To develop the search, we undertook a retrospective analysis of data contained in 200 electronic patient records at one general practice. The records were reviewed to determine the quality, completeness and scope of data that could be retrieved in a search. Search criteria were then designed that drew on clinical indicators from the SPICT™ (Supportive and Palliative Care Indicators Tool) which has been developed and validated to help clinical teams identify patients with advanced heart, liver, kidney, neurological and respiratory disease and cancer [17, 18]. We then tested the search algorithm against all 12,000 adult patient records from two general practices: the practice used initially and a new practice. The results were analysed by the steering committee consisting of a broad range of specialist and generalist palliative care clinicians and GPs and refined for initial testing.

### Phase 2): initial testing

We explored the feasibility and face validity of the computerised search by asking a further 10 general practices across the two participating Health Boards to run the search and then discuss the results in their multi-disciplinary team meeting to see if the search results corresponded with clinical judgement informed by the ‘surprise question’ [19]. The “surprise question” is an empirical question on clinical judgement. If the clinician “would not be surprised if the patient died in the next 6–12 months”, the patient should be assessed for unmet supportive and palliative care needs.

### Phase 3): extended testing

Five general practices in one of the Health Boards then tested the acceptability of the intervention and outcomes of it in actual practice. Each participating general practice was asked to run the search at least twice during a 10–15 week period, review the results at a primary care team meeting observed by the project researcher and select 3–5 patients from the research for care planning. The researcher took notes on the discussion at the meeting to keep a record of actions considered and matters arising. During phase 2 and phase 3, the researcher conducted short interviews with one GP or practice manager at each practice in order to gain feedback about the search’s usefulness.

## Results

### Phase 1) development of the search algorithm

The *search algorithm* represents the program’s criteria for selecting which patients to output as potentially having deteriorating health due to one or more advanced illnesses and a likelihood of unmet supportive and palliative care needs. The algorithm excludes patients already allocated a palliative care code, and is outlined in Table 1. Further details are available from the authors.

**Table 1:** Search algorithm

Search algorithm
Patients were identified if they had any of the following *and had not already been identified for palliative care* (ie already had a palliative care READ code already in place
• A READ code indicating at least one of the following conditions within the last 18 months.
ο Cancer diagnosis
• stomach, pancreas, lung
• any metastatic cancer
ο Chemotherapy or radiotherapy treatment
ο MRC breathlessness - stage 5
ο Chronic kidney disease - stage 5
ο Diuretic resistant ascites
ο Alcoholic and non-alcoholic cirrhosis of the liver
ο Chronic liver disease
ο Housebound patient
ο An Exception made from any of the following Quality Indicators (QoF):
• Heart failure, diabetes, COPD, dementia, stroke, peripheral arterial disease
• Any coding for heart failure cross-referenced with chronic kidney disease - stage 4 or 5 (This combination carries a poor prognosis)

Patients who were already on the practice palliative care register or who had previously been identified with a palliative care code (for example, people currently receiving palliative oncology treatment) were excluded because they were presumed to have already been identified as candidates for palliative care. As an element of the search we developed a proxy for clinical intuition about a patient’s deteriorating health by including “QOF exceptions.” UK general practices are funded to participate in specified healthcare activities listed in “Quality Frameworks” (QoF) [20]. GPs can enter an “exception Read code” for individual patients against specific QoFs if they think that the actions required would be inappropriate for them. This is usually because the patient is considered by their GP to be too ill to benefit from health promotion activities. We were unable to search free-text information due to limitations with the software.

### Phase 2) initial testing

The search identified from 0.5–1.7 % of patients on the practice’s list who were not already entered onto the practice palliative care register as possible candidates for supportive and palliative care in each practice (see Table 2). There was considerable variability between practices in the proportion of patients identified by the search that were also selected by at least one primary care clinician using the ‘surprise question’. This ranged from 21–83 %. The mean proportion of patients identified by both the search and at least one participant was 50 % (median 39 %). At the team meeting, all the participating primary care clinicians could identify at least one patient when prompted by the search results. However, they struggled to suggest concrete actions that could be taken to improve the care of many of those patients. In many cases, patients were considered to have intractable social problems such as a difficult family situation. In others, the primary care team members worried that a “palliative care” approach risked harming the patient because of its association with dying.

**Table 2:** Results of phase 1 practice meetings

ID	Practice size	PCR %	Participants at practice meeting	Number of patients identified by the search	% of patients identified by the search	Number of search patients assessed as surprise positive	Percentage of search patients assessed as surprise positive
1	10329	0.83 %	10 (6 GP, 2 PM, 1PN, 1 IT)	76	0.74 %	51	67 %
2	10775	0.05 %	7 (6 GP, 1 PM)	121	1.13 %	43	36 %
3	10924	0.14 %	12 (5 GP, 2 PM, 3 PN, 1 IT, 1 medical student)	55	0.50 %	37	67 %
4	2960	0.20 %	4 (3 GP, 1 PM)	50	1.69 %	18	36 %
5	13961	0.24 %	7 (7 GP)	90	0.64 %	38	42 %
6	4491	0.02 %	5 (4 GP, 1 PM)	39	0.87 %	32	82 %
7	4471	0.13 %	3 (3 GP)	55	1.23 %	46	83 %
8	12442	0.16 %	10 (10 GP)	117	0.94 %	25	21 %
9	1890	0.26 %	2 (1GP, 1 PM)	23	1.22 %	6	26 %
10	10986	0.37 %	10 (8 GP, 1 PM, 1 IT)	121	1.10 %	43	36 %

*PCR%* initial percentage of patients on the palliative care register; participants (*GP* general practitioner, *PM* practice manager, *IT* information technology expert, *PN* practice nurse)

### Phase 3) extended testing

We tested the practical use and acceptability of the search successfully over time. Five practices were recruited (three continuing from phase 2 and two additional practices). Four practices ran the standardised search twice, each time followed by a practice meeting to review the list of patients generated. The fifth practice completed the process just once. At the practice meeting, the participants were asked to review and create a plan of action for a maximum of 1 patient per GP present; this resulted in between 1–3 patient plans created at each meeting.

The most commonly chosen action when a patient was identified by the search and confirmed as likely to benefit from better coordinated care by the clinicians was starting an anticipatory care plan using the Scottish electronic Key Information Summary (KIS). The KIS is a new form of electronic care planning record introduced throughout Scotland in 2013 (see Table 3). Of the 43 patients reviewed, six were added to the palliative care register and 23 had a KIS started as a result of the identification and review intervention (see Table 4). A variety of other actions (28 in total) were documented for 1–3 patients each including contacting a family carer, discussing end-of-life care and cardio pulmonary resuscitation status, and a review of social care at home.

**Table 3:** Key Information Summary (KIS)

The KIS (Key Information Summary) is a new IT development in NHS Scotland pioneering a shared medical record between healthcare professionals. It allows selected parts of the GP electronic patient record to be shared with other parts of the NHS, using a template within the GP clinical system, and is more efficient and safe than previous paper-based and email-based methods. The level of detail contained on a KIS will depend on the complexity of the patient’s clinical condition, and it is designed to be added to as the patient’s clinical condition progresses. It was introduced in Scotland in 2013, and is an extension to the ECS (Emergency Care Summary). The KIS can contain Read Coded data and free-text. Changesto the KIS entered by the patient’s General Practice are updated to the central KIS every two hours. The central KIS can be accessed by Out of Hours, and some other services e.g. Accident & Emergency, Acute Receiving Unit, and Scottish Ambulance Service. Although other services can read a KIS, only General Practices can (at the time of the project) add information into a KIS. For more information, see http://www.nisg.scot.nhs.uk/why-nisg/our-services/project-management/key-information-summary-kis

**Table 4:** Results of phase 2 practice meetings and actions taken

Action (total number of patients reviewed 43^a^)	Number of times the action was taken
Start a Key Information Summary	23
Add to palliative care register	6
Review medication	1
Schedule home visit	1
Consider power of attorney	4
Continue current treatment	5
Intervene with respect to drugs or alcohol consumption	1
Check current package of care at home	1
Contact family member or informal carer	3
Review current KIS	2
Refer for physiotherapy review	1
Social care review	2
Consider Do Not Attempt Cardio- Pulmonary Resuscitation conversation	2
Arrange respite care	1
Discuss with heart-failure nurse	1
Refer for specialist palliative care	1
Discuss end-of-life care	2
Total number of actions taken	57

^a^Note that in some cases reviewed patients received multiple actions

### Please insert Box 2 Key Information Summary (KIS) here

#### Qualitative results

A total of 19 multidisciplinary meetings were observed and 12 participating primary care team members were interviewed. A thematic analysis [21] of this data found three themes (time, identity and coping) that presented challenges and opportunities to enhancing identification through computer record searching.

##### Time

GPs perceived that if they had to take more time for assessment and care planning actions that they would have less time to spend on other healthcare activities. There would need to be a clear and obvious benefit to patients in order to justify reducing other activities or the service development would need to be time-saving.

##### Identity

GPs were reluctant to label patients as “palliative” due to the association of the term with death and dying. This was a significant barrier to identifying more patients at an earlier stage in their illness trajectory.

##### Coping

Primary care clinicians also felt they were having to cope with conflicting priorities and complex, sometimes intractable, needs. An intervention which potentially helped them cope with these issues would be highly valued.

## Discussion

It is possible to run a search of existing electronic primary care records in the UK to identify patients with deteriorating health. The most popular action by clinicians in response to the search results was to start an electronic anticipatory care plan using the KIS facility and to share this information with other service providers. The search therefore provided an additional resource that could be integrated into routine clinical practice without requiring any new software or hardware or additional practice meetings. However, as the observational data showed, there was resistance among primary care teams to expanding patient identification in this way, and a reluctance to introduce ‘palliative care’ at an earlier stage than currently because of its widespread association with terminal care and connotations around “giving up” and “losing hope.” Finally, GPs expressed doubts about adopting any intervention which could potentially increase their already demanding workload without a direct and obvious benefit for patients.

Several strengths and limitations are apparent in this study. To maximise generalizability, we solely used pre-existing primary care IT resources in a diverse sample of GP practices: rural versus urban, small versus large and those with a high percentage of patients on their palliative care register versus those with a low percentage. However, this limited the search terms that could be used in the algorithm; many clinical indicators used in other disease specific prognostic tools and palliative care identification tools did not exist as Read codes in the patient records or had not been entered consistently in a searchable way. For example, there was no way to search for “frailty” or changes in condition such as declining performance status. There were software-specific limitations to the search: for example, at the time, the Vision GP record system was not able to search through free-text entries in patient records. Finally, due to limitations in the software, it was not possible to indicate in the search result which search term(s) had identified the patient. The search was developed solely in Vision in order to focus on ensuring that the underlying concept worked; future development will need to involve translating the search into other systems. However, as a proof of concept, this project illustrated that computer record search algorithms facilitating identification of patients who might benefit from supportive and palliative care review and planning can be introduced more widely in general practice without requiring new hardware or software or IT re-designs.

A review of other electronic record searching software packages indicates the complexity of the process. The “electronic GSF” being developed in England reported that a huge number of patients could be identified, but their subsequent assessment and care planning was not quantified [22]. A systematic literature review across Europe to identify tools that support identification of patients with palliative care needs found seven but none was in widespread use [14]. The NECPAL (*Necesidades Paliativas*) identified about 1.3 % of the population of Catalonia and 7 % of all people over the age of 65 as meeting its criteria [23]. This is broadly similar to the findings from our intervention. Our research sheds some light on the difficulties of integrating such clinical tools into software algorithms.

## Conclusions

Using electronic record searches in primary care to help identify patients has great potential benefits. However, primary care teams are already facing significant time and workload pressures and the addition of new IT ‘solutions’ depends on demonstrable value in improving individual patient care alongside efficiency for the practice team. Our findings indicate that a tool which facilitates primary care teams in identifying patients for generic needs assessment which can be integrated with holistic, anticipatory care planning would be acceptable to both professionals and service users.

Instead of perpetuating the binary depiction of ‘active treatment’ versus ‘palliative care’, the use of a holistic record such as the KIS allows for earlier anticipatory care planning for people at risk of deteriorating and dying that can be integrated with a palliative care approach over time without requiring any formal “transition’. Recent controversies over the care of patients thought to be at the end-of-life should cause us to consider the benefits and potential harms of identifying relevant individuals, as well as how we can do it better. A badly-designed and implemented computer search would rightly attract negative press attention if it focused on planning for ‘dying’ rather than helping people live as well as possible to the end of their life. On the other hand, an electronic search tool that enables primary care clinicians to identify more patients who might benefit from anticipatory care planning and regular review could help address current inequities in the provision of supportive and palliative care in the community, and lessen the risk of under-informed decision-making during an acute episode of illness.
